# mRNA Vaccines against SARS-CoV-2: Advantages and Caveats

**DOI:** 10.3390/ijms24065944

**Published:** 2023-03-21

**Authors:** Miriam Echaide, Luisa Chocarro de Erauso, Ana Bocanegra, Ester Blanco, Grazyna Kochan, David Escors

**Affiliations:** Oncoimmunology Unit, Instituto de Investigación Sanitaria de Navarra (IdiSNA), Navarrabiomed-Fundación Miguel Servet, Universidad Pública de Navarra (UPNA), Hospital Universitario de Navarra (HUN), 31008 Pamplona, Spain; mechaidg@navarra.es (M.E.); luisa.chocarro.deearuso@navarra.es (L.C.d.E.); ana.bocanegra.gondan@navarra.es (A.B.); ester.blanco.palmeiro@navarra.es (E.B.)

**Keywords:** SARS-CoV-2, mRNA vaccines, BNT162b2, mRNA-1273, Comirnaty, Spikevax, variants, myocarditis, Th17 response

## Abstract

The application of BNT162b2 and mRNA-1273 vaccines against SARS-CoV-2 infection has constituted a determinant resource to control the COVID-19 pandemic. Since the beginning of 2021, millions of doses have been administered in several countries of North and South America and Europe. Many studies have confirmed the efficacy of these vaccines in a wide range of ages and in vulnerable groups of people against COVID-19. Nevertheless, the emergence and selection of new variants have led to a progressive decay in vaccine efficacy. Pfizer–BioNTech and Moderna developed updated bivalent vaccines—Comirnaty and Spikevax—to improve responses against the SARS-CoV-2 Omicron variants. Frequent booster doses with monovalent or bivalent mRNA vaccines, the emergence of some rare but serious adverse events and the activation of T-helper 17 responses suggest the need for improved mRNA vaccine formulations or the use of other types of vaccines. In this review, we discuss the advantages and limitations of mRNA vaccines targeting SARS-CoV-2 focusing on the most recent, related publications.

## 1. Introduction

**Figure 1:**
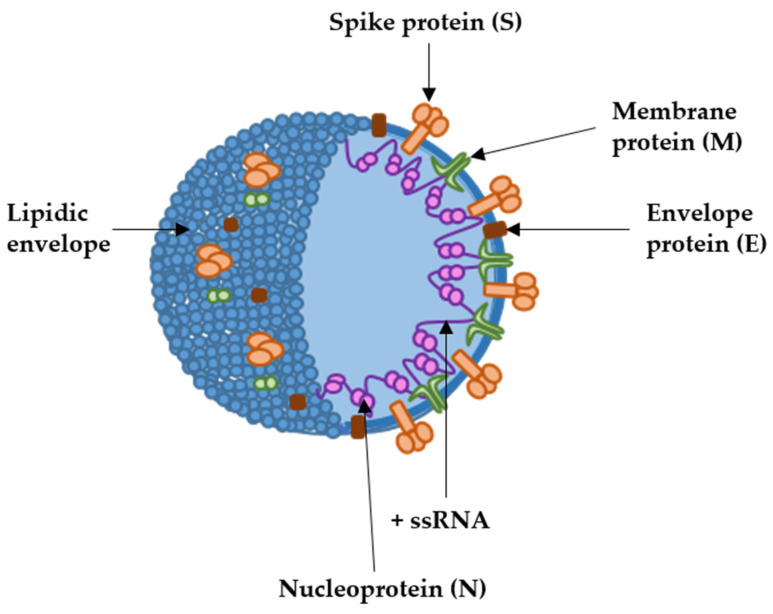
SARS-CoV-2 structure with the localization of the main structural proteins (arrows). The coronavirion is spherical, of about 100 to 120 nm of diameter. +ssRNA, indicates the positive-sense single-stranded RNA genome.

Coronaviruses comprise a group of pathogens affecting many vertebrate species, from birds to humans [1]. These viruses generally cause respiratory and enteric diseases, but some coronavirus species can cause other diseases such as hepatitis or encephalitis in non-human vertebrates. Coronaviruses are single-stranded positive-sense RNA (+ssRNA) viruses belonging to the *Coronaviridae* family [2]. Their genome encodes replicase/transcriptase proteins, structural proteins and a set of non-structural proteins linked to their virulence and proofreading activities of the replicase complex [3,4]. The coronavirus virion contains a helical nucleoprotein made of a single +ssRNA genome bound to the nucleocapsid protein (N) [5]. This nucleoprotein is further organized into a packed internal core, enveloped by the virus membrane which derives from the endoplasmic reticulum/Golgi. The spike (S), membrane (M) and envelope (E) proteins are the main structural proteins inserted into the virus envelope [6]. The spike protein is further organized into trimers, forming the “corona” of peplomers that gives rise to the name of the family (Figure 1). In 2003, an outbreak of infectious pneumonia put the international community into high alert, leading to the discovery of severe acute respiratory syndrome coronavirus-1 (SARS-CoV-1), the first human pathogen of the family to cause lethal disease [7,8]. That first outbreak was controlled until a second one caused by a closely related coronavirus, SARS-CoV-2, originated one of the most severe pandemics in human history. The global epidemic was so dramatic that it accelerated the engineering of vaccines targeting SARS-CoV-2 at rates never seen before. The previous experience with other coronaviruses, including SARS-CoV-1 and MERS-CoV [9,10,11], prompted the selection of the spike protein as the main immunogen in most vaccines [12,13]. From the four main structural proteins, the S protein is one of the most immunogenic, at least in raising neutralizing antibody responses. Among all the potential types, mRNA vaccines soon became the primary candidates.

Vaccines based on antigen delivery through mRNA are produced by a simple and fast procedure which consists of the amplification of the RNA nucleotide sequence encoding the open reading frame of the desired gene, plus further modifications to enhance stability and translation. The procedure is performed in automatized factories in which the risk of contamination with unrelated material is very low [14].

The first mRNA vaccine for infectious diseases was developed against influenza A in 2012. In vivo experiments in mice showed specific B- and T-cell based protection. The efficacy of the vaccine was also proven in ferrets and pigs [15]. After that, various mRNA vaccines were tested in animal models to evaluate their efficacy against the Zika virus, Ebola virus, cytomegalovirus and human immunodeficiency virus (VIH), among others [16,17,18,19,20,21].

Several vaccines have been developed for SARS-CoV-2 to reduce its transmission and virulence. Among other adenoviral and protein-based vaccines, the European Medicines Agency (EMA) authorized two mRNA vaccines for human use, BNT162b2/Pfizer–BioNTech and mRNA-1273/Moderna, in December 2020 and January 2021, respectively.

The BNT162b2 vaccine consists of a lipid nanoparticle, which contains an mRNA encoding the full-length spike protein with two proline substitutions (in positions 986 and 987) in the S2 subunit to maintain the protein in the prefusion conformation [22,23]. Similarly, the mRNA-1273 vaccine consists of a lipid nanoparticle capsule constituted by four lipids, which also carries an mRNA encoding the SARS-CoV-2 full-length spike glycoprotein with the intact furin cleavage site and the two proline substitutions in the S2 subunit [24,25]. Up to three doses of these vaccines have been allowed and even four doses in vulnerable people at risk [26,27,28].

The lipid nanoparticle (LNP) of BNT162b2 is composed of ALC-0159 (2[polyethylene glycol)-2000]-N,N-ditetradecylacetamide) and DSPC (1,2-distearoyl-sn-glycero-3-phosphocholine) which play an important role in the formation of a stable lipid-bilayer nanoparticle. The LNP is structurally supported by cholesterol. Finally, the ALC-0315 ((4-hydroxybutyl) azanediyl)-bis(hexane-6,1-diyl)-bis(2-hexyldecanoate)) is the fundamental component for mRNA delivery into the cell. In addition, the vaccine is supplemented with salt buffers in order to balance the pH, and sucrose to protect the vaccine during freezing. Similarly, the mRNA-1273 LNP is stabilized by polyethylene glycol (PEG) 2000 DMG and DSPC, which form a lipid bilayer that is structurally supported by cholesterol. In contrast to BNT162b2 LNP, it carries lipid SM-102 in order to release the mRNA into the cell. This vaccine is also supplemented with salt buffers to balance the pH and with sucrose that serves as cryo-protectant.

LNP based-vaccines present a challenge due to the lack of thermostability and ultra-cold storage requirements, a fact that has limited their use in resource-poor countries. In addition, lipid and cholesterol excipients make the vaccines prone to oxidative degradation, which could decrease the stability of the vaccines. Furthermore, these BNT162b2 and mRNA-1273 mRNA vaccines, in particular, could pose a challenge in terms of delivery due to the long ribonucleic acids (4284 and 4004 nucleotides, respectively) with a number of modified nucleosides.

The BNT162b2 vaccine has been approved in 85 countries from North and South America and Europe; while mRNA-1273 has been distributed in 45 countries in Europe and North America [29].

Millions of doses of these mRNA vaccines have been administered worldwide during the pandemic. This has allowed the consolidation of the efficacy and safety data on these vaccines, and confirmed the decay in efficacy associated with the relatively short duration of protection. Data on protection against SARS-CoV-2 emerging variants have also been obtained. Here, we discuss the advantages and limitations of these mRNA vaccines and the recent adaptations approved for SARS-CoV-2 Omicron variants.

## 2. Advantages and Caveats of Efficacy and Safety of mRNA SARS-CoV-2 Vaccines

The advantages of the BNT162b2 and mRNA-1273 vaccines in terms of efficacy were readily noticeable right at the beginning of their administration to the general population. The application of these vaccines was quickly associated with a decrease in COVID-19 symptomatology and spread [30]. Their fast efficacy was caused by a combination of factors: the induction of high titers of neutralizing antibodies, the activation of T-cell responses, and a demonstrated efficacy within different population groups, including vulnerable people such as the elderly.

In contrast, over the course of time, we have been aware of some of their limitations, especially after the selection and propagation of variants. The most significant are the rare but serious adverse events specifically associated with these mRNA vaccines, short-lived protection, reduced efficacy towards variants of concern and the activation of Th17 immune responses which can exacerbate inflammatory reactions.

### 2.1. Induction of Neutralising Antibodies and T-Cell Activation

In phase three of their respective clinical trials, vaccination with BNT162b2 and mRNA-1273 vaccines provided protection against symptomatic COVID-19 in 95 and 94.1% of the vaccinated participants, respectively. These trials were carried out in groups of subjects with ages ranging from 16 to 55. Their efficacy was also proven in older adults comprising the population most vulnerable to COVID-19 (>65 years of age) and also in adolescents (<16 years of age) [23,24,31,32,33]. Further studies confirmed the efficacy of these vaccines against the SARS-CoV-2 original strain by the fast induction of high titers of IgM and IgG antibodies specific towards the S protein, and with potent neutralizing capacities. These antibody titers remained detectable up to six months post-vaccination. Additionally, some studies evaluated the generation of T-cell responses towards S-derived peptides, demonstrating the presence of S-specific CD4 and CD8 T cells within 10 days to 9 weeks following the first and second dose, or even up to 6 months post-vaccination in healthy donors [29,34,35,36,37,38,39,40,41,42]. Furthermore, third and fourth doses led to improved immune responses compared to two doses of mRNA vaccines, leading to a peak in IgG titers in the fourth week postvaccination [43,44,45]. 

### 2.2. Efficacy in Vulnerable Populations

However, it turned out that not all vulnerable groups of people benefit from the current mRNA vaccines. This is specially the case for organ-transplanted patients or patients suffering multiple sclerosis. These patients did not benefit from BNT162b2 and mRNA-1273 vaccines due to their immunosuppressive treatments [46,47,48,49,50,51,52,53]. On the other hand, the efficacy of these vaccines has been demonstrated in patients with several types of cancers. Most studies highlight the induction of S-specific antibodies after mRNA vaccination in solid-tumor patients and oncohematological patients. This is especially true in the third week after the administration of the second dose, reaching similar numbers of antibody titers as healthy donor groups [54,55,56,57,58,59,60]. Some studies also detected CD4 and CD8 T cells specific for the S protein in solid-tumor patients up to 6 months post-vaccination. In these latter cases, the antibody titers were comparable to those achieved in healthy donors vaccinated with the mRNA vaccines [42,61,62,63]. Nevertheless, patients with hematological cancers and vaccinated with the mRNA vaccines presented decreased numbers of specific-T cells compared to healthy individuals [63,64]. The specific studies are summarized in Table 1.

### 2.3. Duration of Protection

As reported by several studies, S-specific IgGs induced by mRNA vaccines decrease 6 months after the second dose of mRNA vaccination [42,65]. As mentioned previously, the third and fourth dose further significantly increase IgG titers compared to titers achieved in subjects vaccinated with only two doses; however, IgG titers again decrease six months after the booster dose [44,66]. It needs to be highlighted that in patients with cancer, the persistence of antibody responses is generally shorter compared to healthy subjects following vaccination [67,68].

**Figure 2:**
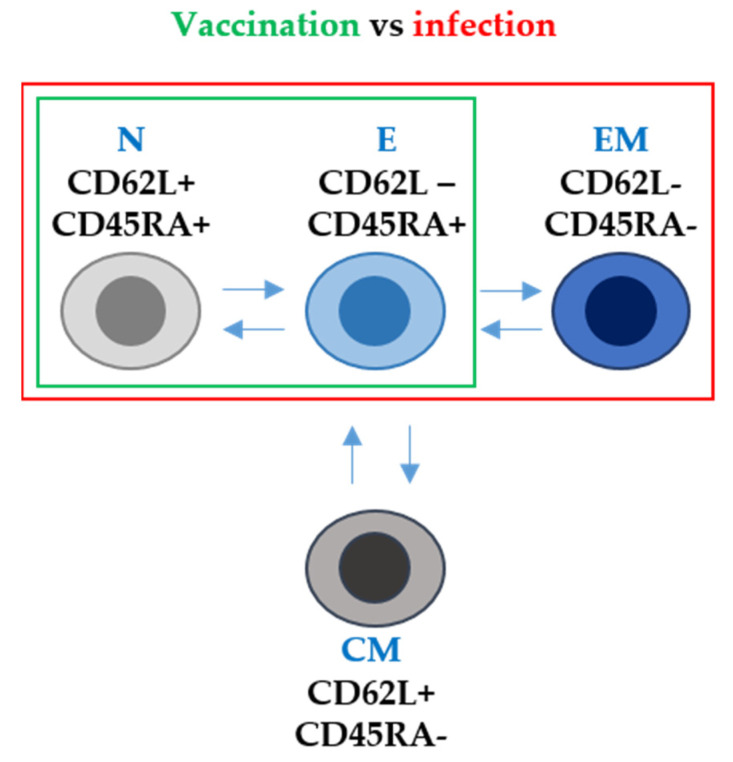
T-cell phenotype after mRNA vaccination and after SARS-CoV-2 infection in terms of CD62L and CD45RA surface marker expression. N, E, EM and CM stand for naïve, effector, effector memory and central memory T-cell subsets. Arrows indicate the differentiation pathways between the different T-cell phenotypes. Within the green box, T-cell subsets differentiated following mRNA vaccination only. Within the red box, T-cell subsets differentiated following infection with SARS-CoV-2.

According to the duration of T cell responses, some studies reported the expansion of vaccine-specific T cells with a stem cell memory phenotype (T_SMC_). This is an important observation, because these T cells could persist for decades, providing long-term protection against SARS-CoV-2. However, in general terms, the specific CD4 and CD8 T cells are generally lost 6 months post-vaccination [69]. T-cell responses can be studied in more detail by analysing the phenotype of T cells expanded following vaccination with the mRNA vaccines. For example, CD62L and CD45RA expression in T cells was assessed by us in a recent study [42]. CD62L and CD45RA surface markers are involved in lymphocyte migration to inflammation sites and participate in T-cell receptor (TCR) signal transduction during antigen recognition [70]. In human T cells, these markers can be used to identify four types according to their differentiation degree: naïve (CD62L+ CD45RA+), central memory (CD62L+ CD45RA^neg^), effector memory (CD62L^neg^ CD45RA^neg^) and effector T cells (CD62L^neg^ CD45RA^neg^) [71,72]. Our study reported that both healthy individuals and patients with cancer without previous SARS-CoV-2 infection showed an expansion of effector T cells (CD62L- CD45RA+) after mRNA vaccination. However, importantly, these mRNA vaccines did not expand T cells with an effector-memory phenotype (CD62L- CD45RA-). This is in stark contrast to vaccination of individuals who had had a previous SARS-CoV-2 infection. In these subjects, vaccination achieved the expansion of effector memory T cells [42] (Figure 2).

### 2.4. Activation of the T-Helper 17 Responses

**Figure 3:**
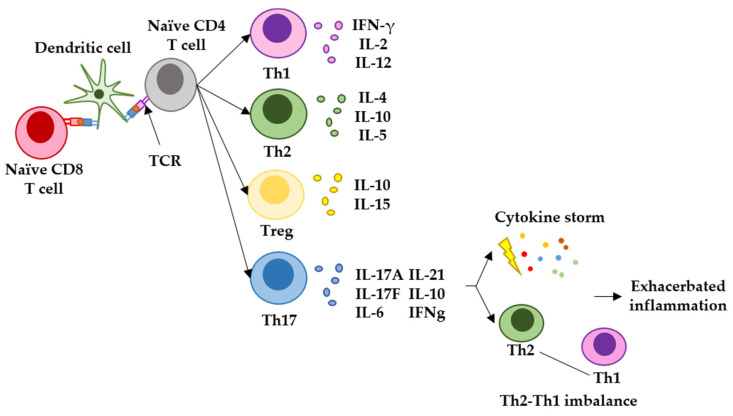
Representation of the main T-helper responses after antigen presentation to CD4 T cells and the role of Th17 response in generating exacerbated inflammation. Naïve CD4 and CD8 T cells recognize antigenic peptides presented by dendritic cells through the T-cell receptor (TCR). Different T-helper pathways can be activated, but the main responses observed in infectious diseases and vaccination are represented here. The Th1 response is characterized by T cells expressing IFN-gamma and IL-2, and regulate antiviral cytotoxic responses. The Th2 response is characterized by T cells expressing mainly IL-4 and IL-10, and regulated B-cell maturation to plasma cells, leading to antibody responses and airway inflammation. T-regulatory cells express mainly IL-10 and IL-15, and inhibit autoreactive damage. Finally, the Th17 response is characterized by T cells expressing mainly IL-17, IL-6, IL-21, IL-10 and IFNgamma. Th17 responses are strong inflammatory reactions caused by an immunological stress, and can lead to cytokine storms and imbalance in Th1-Th2 responses.

In many cases, SARS-CoV-2 leads to the death of the patient by exerting an exacerbated inflammatory response within the lungs of infected patients. Some studies have linked the establishment of a Th17-type of T-cell response during COVID-19 with the activation of a pro-inflammatory cytokine cascade (cytokine storm) [73,74]. For most vaccines targeting infectious agents, it would be desirable to elicit immune responses of the Th1 and Th2 types. These responses are efficacious in raising antiviral immunity while activating antibody responses. Th1 responses are regulated by T cells which mainly express IFN-gamma and IL-2, and they have a key role in attracting immune cells to the site of infection and in mediating the T-cell cytotoxicity of infected cells; Th2 responses are regulated by T cells expressing mainly IL-4 and IL-10, and they are involved in efficacious antibody production and airway inflammation observed in some respiratory diseases [75]. On the other hand, Th17 responses are regulated by T cells expressing IL-17, IL-6 and IFN-gamma. Th17 responses are fast, strong inflammatory reactions which can be critical in situations of high immunological stress. However, Th17 responses imbalance Th1-Th2 immunity, contributing to the exacerbation of inflammation, and in the case of SARS-CoV-2, its pathogenesis [73,75,76,77]. Recent studies have reported the induction of elevated concentrations of IL-17 after mRNA vaccination, indicating that mRNA vaccines trigger this strong inflammatory response [78] (Figure 3). Indeed, our study described an enhancement of this response in vaccinated oncologic patients without previous SARS-CoV-2 infection [42]. These results indicated that mRNA vaccination in patients with cancer can potentiate their chronic inflammatory status often originated and exacerbated by solid tumors, or their treatments [79,80,81,82,83,84,85].

### 2.5. Loss of Efficacy towards Variants of Concern

**Figure 4:**
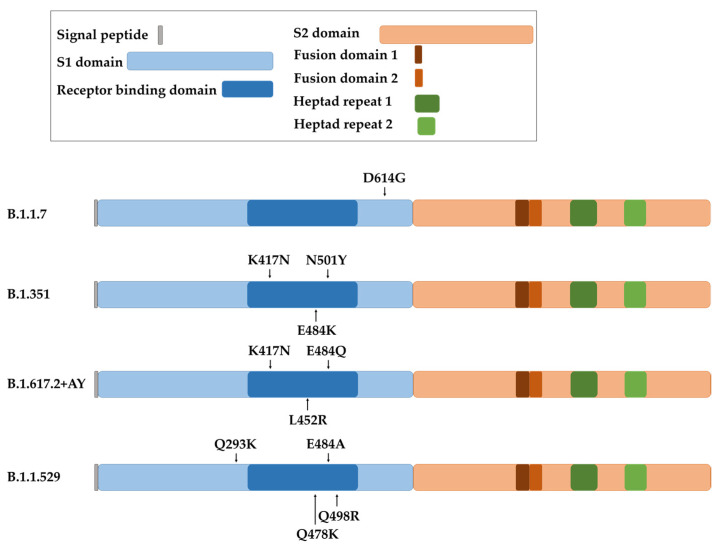
Representation of the spike protein of SARS-CoV-2 with the indicated domains and the localization of the main mutations involved in increased virus transmission for each variant (B.1.1.7 or Alpha variant, B.1.351 or Beta variant, B.1.617.2 + AY or Delta plus variant, and B.1.1.529 or Omicron variant).

The coronavirus S protein is the largest and most exposed antigen of the viral particle. Three molecules of the S protein form the coronavirion peplomer, which confers entry to the cell and tissue tropism [4,86]. For SARS-CoV-2, the receptor for the S protein is the ACE2 surface protein [87]. This fact makes the S gene subject to strong selective pressure from the immune system, which leads to viral escape mechanisms by increasing the number of mutations, specially concentrated in the proximities of the receptor-binding domain (RBD) (Figure 4). As most SARS-CoV-2 vaccines utilize the S protein sequence from the original Wuhan strain, these escape mutants can also escape from immune responses caused by the vaccines. This, in turn, results in a subsequent decrease in efficacy for all vaccines which use the original S protein sequence.

The first dominant D614G substitution in the spike protein arose in the B.1.1.7 variant, more commonly known as Alpha SARS-CoV-2 variant (Figure 4). It has to be noted that this mutation is outside the RBD, but it nevertheless increased viral replication and transmission. Several studies later demonstrated that the D614G mutation did not decrease the protection conferred by mRNA-vaccines, which was maintained at a 94–95% of efficacy and generating comparable titers of neutralizing antibodies compared to the efficacy towards the original Wuhan strain [88,89]. Several other mutations were selected. For example, E484K, N501Y and K417N mutations in the B.1.351 variant, also known as Beta (Figure 4). These mutations were reported to cause a decline in efficacy of mRNA vaccines. The neutralizing capacities of sera from mRNA-1273- and BNT162b2-vaccinated individuals was approximately 10-fold lower towards this variant compared to the original strain [90,91]. Even so, mRNA vaccines continued to be effective against the spreading and pathogenesis of SARS-CoV-2. This was in contrast to the adenoviral-vectored ChAdOx1 vaccine, which was associated with a significant decrease in efficacy against this variant [92].

The COVID-19 strain B.1.617.2 (Delta) contained 18 novel mutations compared to the original strain [93] (Figure 4). These changes increased the transmission rate of the virus and increased its affinity to lung epithelial cells [94]. In particular, E484Q and L452R mutations enhanced immunological evasion and resistance to neutralizing antibodies from vaccinated individuals and convalescent people [95]. The protective efficacy of mRNA vaccines decreased to 88% for this variant [96], with a subsequent decrease in protection against infection six months post-vaccination [97]. In addition, the B.1.617.2 + AY sub-variants (Delta plus) selected an extra mutation (K417N) which potentiated escape from neutralizing antibodies generated by the original vaccines [94,98]. Moreover, a recent comparative study of S mutations in Alpha, Beta and Delta variants highlighted the progressive capacity of the virus strains to enter cells independently of S protein–ACE2 interactions. This fact augments transmissibility of the virus as the number of mutations increases [99]. This situation is not novel with coronaviruses, as it is likely that some coronavirus species can use a co-receptor to modulate the in vivo tissue tropism [86,100,101,102].

A variant of high interest was selected in regions with a high percentage of vaccinated population, suggesting that this variant was an escape mutant from the vaccines themselves. This variant was termed B.1.1.529, or Omicron, and its S gene accumulated more than 30 mutations compared to the original strain [98,103] (Figure 4). T478K, Q293K, Q498R and E484A contributed to an elevated transmission rate and evasion from neutralizing antibodies [104]. Due to this enhanced escaping capacity, the protection achieved with BNT162b2 and mRNA-1273 vaccines decreased to 30% after three doses, and to 47.2% in older adults [93,105,106,107,108,109,110,111,112]. This variant is still evolving, leading to Omicron sub-variants such as BQ, XBB and BF.7, with high capacities to avoid neutralizing antibodies elicited by the original vaccines [108,113].

The decline in the protection of the population against SARS-CoV-2 caused by the selection of new variants has prompted the redesign of mRNA vaccines. This is the advantage of mRNA vaccines, which allow fast modifications by just changing the immunogenic transgene to target variants. Pfizer–BioNTech and Moderna brought to the market two bivalent vaccines—Comirnaty and Spikevax—containing mRNAs encoding the spike protein of the original variant together with BA.4-5 Omicron variants [114,115]. Booster doses with these vaccines seem to offer increased protection against new Omicron subvariants, generating higher titers of Omicron-specific neutralizing antibodies than monovalent vaccines [115,116]. Nevertheless, long-term follow-up studies should be carried out to obtain more solid and robust data on the impact on the protection and spreading of the virus in the human population.

### 2.6. Adverse Events Caused by mRNA Vaccines

It has to be remarked that no serious adverse effects were described in the clinical trials assessing mRNA vaccines BNT162b2 and mRNA-1273 which led to their approval [22,33]. However, the administration of millions of vaccine doses has uncovered rare adverse events and complications, characterized by a diversity of symptoms. In general terms, complications from SARS-CoV-2 infection outweigh the risk of suffering these rare adverse effects following vaccination. Nevertheless, it is necessary to follow the evolution of the affected population to identify causal agents of adverse events to either improve vaccine formulations, or to better allocate the populations that need vaccination.

Two large-scale studies were carried out in the United Kingdom in about 40 million people vaccinated with sequential doses of the adenovirus-based ChAdOx1 vaccine or mRNA vaccines to evaluate cardiac adverse events. The results showed the occurrence of myocarditis in 0.004 and 0.007% of the vaccinated people with ChAdOx1 and mRNA vaccines, respectively. A statistical analysis of the data in both studies uncovered an increased risk of suffering myocarditis after the first dose of ChAdOx1 and BNT162b2 vaccine than after the further booster doses of the mRNA-1273 vaccine [117,118]. These studies indicated that especially males under 40 years of age had an elevated risk [119]. A study carried out in the USA over large-scale databases reported an elevated occurrence of myocarditis or pericarditis in mRNA-vaccinated people between 18 and 25 years of age following the second dose, without significant differences between BNT162b2 and mRNA-1273 vaccine formulations [120]. On the other hand, no clear association between vaccination and cardiac arrhythmia has been demonstrated, as most cases occurred after SARS-CoV-2 infection and this can be a confounding factor [117]. These adverse events have been therefore stated in the official websites of Comirnaty and Spikevax and by the European Medicine Agency (EMA), more specifically occurrence of myocarditis and pericarditis in some vaccinated people (https://www.comirnaty.com/, https://spikevax.com/, https://www.ema.europa.eu/en/documents/prac-recommendation/signal-assessment-report-myocarditis-pericarditis-spikevax-previously-covid-19-vaccine-moderna-covid_en.pdf, https://www.ema.europa.eu/en/documents/prac-recommendation/signal-assessment-report-myocarditis-pericarditis-spikevax-previously-covid-19-vaccine-moderna-covid_en.pdf; accessed on 20 March 2023). Isolated cases of vasospastic angina and Takotsubo cardiomyopathy have also been observed after mRNA vaccination. In addition, there are reported cases of myocardial infarction, stroke and pulmonary embolism in people older than 75 years of age after BNT162b2.

To date, although with less solid data, other consequences associated with the vaccines have been detected and reported. For example, alterations in the menstrual cycle such as abnormal bleeding and delayed menstruation following the second booster dose of the BNT162b2 vaccine [121,122]. Studies reporting the main adverse events are summarized in Table 2.

Interestingly, recent data have reported that consuming alcohol, tobacco or drugs, apart from decreasing the humoral response generated by BNT162b2 mRNA vaccine, also activates the ACE2 receptor enhancing the “spike effect” of COVID-19 vaccines. The “spike effect” refers to the interaction of the endogenous spike protein with the ACE2 receptor, resembling the COVID-19 pathology and leading to rare neurological complication, such as Guillain–Barre syndrome and Bell’s palsy.

## 3. Conclusions

The use of mRNA vaccines targeting the spike protein of SARS-CoV-2 has constituted one of the main barriers in the battle against this pandemic. It is estimated that SARS-CoV-2 vaccines, including those based on adenovirus vectors, protein-based vaccines and mRNA vaccines have saved the lives of around 20 million people worldwide [123]. Even so, it is necessary to review all the updated information on the efficacy and safety of these vaccines during the development and evolution of the pandemic. It is also important to evaluate the changes in vaccine policy, now that a very large number of people have been infected with SARS-CoV-2 and present a degree of protection. Indeed, the frequent administration of booster doses due to the loss of post-vaccination immunity is causing some experts to warn about a possible link between immune exhaustion and frequent vaccination [124]. There is some experimental evidence pointing towards this direction, as observed in a recent study of cancer patients following three doses of BNT162b2 [125].

In addition, the increasing number of mutations within the S protein of emerging variants and the progressive decrease in efficacy of mRNA vaccines suggest that vaccine formulations should be changed by including additional viral targets. For example, some studies including our own have highlighted the role of the N and M proteins of the SARS-CoV-2 virus in the generation of notable antibody and cellular responses [42]. The advantage of incorporating these viral immunogens is that they are subject to lower selective pressure compared to the S protein. Their mutational burden is by comparison much lower than that of the S protein. Hence, using these virus structural proteins could lead to more robust and durable immune responses without the need for regularly changing the S protein strain in vaccine formulations [126].

It also needs to be stressed that improved vaccine formulations should lead to activation of Th1 and Th2 responses to the detriment of Th17 responses. This could circumvent the exacerbated inflammation caused after vaccination which could be linked to serious adverse effects. In addition to vaccine formulation, the fact that these vaccines contain long ribonucleic acids with a number of modified nucleosides, could result in the preparation of batches with identical properties. This, together with the lack of thermostability, suggests that protein-based vaccines are a preferable option to avoid these challenges.

Using other types of vaccines, such as viral-vectored vaccines or protein-based vaccines, or forgoing booster doses, should be considered for people who are predisposed to suffer cardiac adverse effects, especially during periods of the active spread of new variants of the SARS-CoV-2 virus.

## Figures and Tables

**Table 1:** Detectable immune responses in different studies performed in either healthy or oncologic participants after mRNA vaccination.

Participants	Age	Vaccine	Detected Immune Responses	Ref
Healthy	18–55	mRNA-1273	S-specific neutralizing IgGs S-specific Th1 responses	[24]
Healthy	18–55 65–85	BNT162b2	S-specific neutralizing IgGs	[23]
Healthy	12–15	BNT162b2	S-specific neutralizing IgGs	[31]
Healthy	12–17	mRNA-1273	S-specific neutralizing IgGs	[32]
Healthy	18–83	BNT162b2-boost	S-specific neutralizing IgGs S-specific Th1 responses and CD8 T cells	[34]
Healthy	18–55	BNT162b2	S-specific neutralizing IgGs S-specific CD4 and CD8 T cells	[38]
Healthy Naïve and SARS-CoV-2 recovered	22–67	BNT162b2 mRNA-1273	S-specific neutralizing IgGs S-specific CD4 and CD8 T cells Memory B cells	[61]
Solid tumor and oncohematological patients	64–80	BNT162b2	S-specific neutralizing IgGs IFNg-secreting T cells	[63]
Multiple myeloma (MM) and chronic lymphocytic leukemia (CLL) patients	35–81	BNT162b2 mRNA-1273	S-specific serological responses Specific CD8-T-cell responses especially in MM patients	[54]
CLL patients	37–93	BNT162b2 mRNA-1273	Treatment-dependent serological response	[57]
CLL patients	41–88	BNT162b2-boost	Treatment-dependent serological response	[60]
Solid-tumor patients	35–87	BNT162b2	S-specific serological responses	[59]
Solid-tumor patients Naïve and SARS-CoV-2 recovered	40–85	BNT162b2	S-specific IgGs S-specific CD4 and CD8 T cells T-cell phenotypes Myeloid subpopulations	[42]

**Table 2:** Adverse effects and the associated risk factors observed after mRNA vaccination in different studies.

Vaccine	Adverse Effect	Higher Risk Associations Observed	Ref
BNT162b2, mRNA-1273, ChAdOx1	Myocarditis (in 0.004% of vaccinated participants)	After first dose of BNT162b2 or ChAdOx1. After second dose of mRNA-1273.	[117]
BNT162b2, mRNA-1273, ChAdOx1	Myocarditis (in 0.007% of vaccinated participants)	After first dose of BNT162b2 or ChAdOx1 After second dose of mRNA-1273. In males under 40 years of age.	[118]
BNT162b2, mRNA-1273	Myocarditis or pericarditis (rates/million doses: 12.59 and 35.5)	After second dose of mRNA-1273. In males from 18 to 39 years of age.	[119]
BNT162b2, mRNA-1273	Myocarditis and/or pericarditis (in 0.003% of vaccinated participants)	After second dose of mRNA-1273. In males from 18 to 25 years of age.	[120]
Spikevax	Myocarditis and pericarditis (cases/million doses: 253 and 533)	In males from 18 to 29 years of age.	
BNT162b2	Menstrual cycle symptoms: irregular bleeding (in 23.3% of vaccinated participants), dysmenorrhea (in 68% of vaccinated participants), mood changes (in 9.6% of vaccinated participants)	-	[121]

## Data Availability

This manuscript does not contain data.

## References

[1] Gonzalez J.M., Gomez-Puertas P., Cavanagh D., Gorbalenya A.E., Enjuanes L. (2003). A comparative sequence analysis to revise the current taxonomy of the family Coronaviridae. Arch. Virol..

[2] Almazan F., Gonzalez J.M., Penzes Z., Izeta A., Calvo E., Plana-Duran J., Enjuanes L. (2000). Engineering the largest RNA virus genome as an infectious bacterial artificial chromosome. Proc. Natl. Acad. Sci. USA.

[3] Ortego J., Escors D., Laude H., Enjuanes L. (2002). Generation of a replication-competent, propagation-deficient virus vector based on the transmissible gastroenteritis coronavirus genome. J. Virol..

[4] Almazan F., Dediego M.L., Galan C., Escors D., Alvarez E., Ortego J., Sola I., Zuniga S., Alonso S., Moreno J.L. (2006). Construction of a severe acute respiratory syndrome coronavirus infectious cDNA clone and a replicon to study coronavirus RNA synthesis. J. Virol..

[5] Escors D., Camafeita E., Ortego J., Laude H., Enjuanes L. (2001). Organization of two transmissible gastroenteritis coronavirus membrane protein topologies within the virion and core. J. Virol..

[6] Escors D., Ortego J., Laude H., Enjuanes L. (2001). The membrane M protein carboxy terminus binds to transmissible gastroenteritis coronavirus core and contributes to core stability. J. Virol..

[7] Holmes K.V., Enjuanes L. (2003). Virology. The SARS coronavirus: A postgenomic era. Science.

[8] Ksiazek T.G., Erdman D., Goldsmith C.S., Zaki S.R., Peret T., Emery S., Tong S., Urbani C., Comer J.A., Lim W. (2003). A novel coronavirus associated with severe acute respiratory syndrome. N. Engl. J. Med..

[9] Gutierrez-Alvarez J., Honrubia J.M., Fernandez-Delgado R., Wang L., Castano-Rodriguez C., Zuniga S., Sola I., Enjuanes L. (2021). Genetically Engineered Live-Attenuated Middle East Respiratory Syndrome Coronavirus Viruses Confer Full Protection against Lethal Infection. mBio.

[10] Castano-Rodriguez C., Honrubia J.M., Gutierrez-Alvarez J., DeDiego M.L., Nieto-Torres J.L., Jimenez-Guardeno J.M., Regla-Nava J.A., Fernandez-Delgado R., Verdia-Baguena C., Queralt-Martin M. (2018). Role of Severe Acute Respiratory Syndrome Coronavirus Viroporins E, 3a, and 8a in Replication and Pathogenesis. mBio.

[11] Tu C., Crameri G., Kong X., Chen J., Sun Y., Yu M., Xiang H., Xia X., Liu S., Ren T. (2004). Antibodies to SARS coronavirus in civets. Emerg. Infect. Dis..

[12] Long Q.X., Liu B.Z., Deng H.J., Wu G.C., Deng K., Chen Y.K., Liao P., Qiu J.F., Lin Y., Cai X.F. (2020). Antibody responses to SARS-CoV-2 in patients with COVID-19. Nat. Med..

[13] Li K., Huang B., Wu M., Zhong A., Li L., Cai Y., Wang Z., Wu L., Zhu M., Li J. (2020). Dynamic changes in anti-SARS-CoV-2 antibodies during SARS-CoV-2 infection and recovery from COVID-19. Nat. Commun..

[14] Janowski M., Andrzejewska A. (2022). The legacy of mRNA engineering: A lineup of pioneers for the Nobel Prize. Mol. Therapy. Nucleic Acids.

[15] Petsch B., Schnee M., Vogel A.B., Lange E., Hoffmann B., Voss D., Schlake T., Thess A., Kallen K.J., Stitz L. (2012). Protective efficacy of in vitro synthesized, specific mRNA vaccines against influenza A virus infection. Nat. Biotechnol..

[16] Pardi N., Hogan M.J., Pelc R.S., Muramatsu H., Andersen H., DeMaso C.R., Dowd K.A., Sutherland L.L., Scearce R.M., Parks R. (2017). Zika virus protection by a single low-dose nucleoside-modified mRNA vaccination. Nature.

[17] Meyer M., Huang E., Yuzhakov O., Ramanathan P., Ciaramella G., Bukreyev A. (2018). Modified mRNA-Based Vaccines Elicit Robust Immune Responses and Protect Guinea Pigs From Ebola Virus Disease. J. Infect. Dis..

[18] Rauch S., Jasny E., Schmidt K.E., Petsch B. (2018). New Vaccine Technologies to Combat Outbreak Situations. Front. Immunol..

[19] John S., Yuzhakov O., Woods A., Deterling J., Hassett K., Shaw C.A., Ciaramella G. (2018). Multi-antigenic human cytomegalovirus mRNA vaccines that elicit potent humoral and cell-mediated immunity. Vaccine.

[20] VanBlargan L.A., Himansu S., Foreman B.M., Ebel G.D., Pierson T.C., Diamond M.S. (2018). An mRNA Vaccine Protects Mice against Multiple Tick-Transmitted Flavivirus Infections. Cell Rep..

[21] Pardi N., LaBranche C.C., Ferrari G., Cain D.W., Tombacz I., Parks R.J., Muramatsu H., Mui B.L., Tam Y.K., Kariko K. (2019). Characterization of HIV-1 Nucleoside-Modified mRNA Vaccines in Rabbits and Rhesus Macaques. Mol. Therapy. Nucleic Acids.

[22] Polack F.P., Thomas S.J., Kitchin N., Absalon J., Gurtman A., Lockhart S., Perez J.L., Perez Marc G., Moreira E.D., Zerbini C. (2020). Safety and Efficacy of the BNT162b2 mRNA COVID-19 Vaccine. N. Engl. J. Med..

[23] Walsh E.E., Frenck R.W., Falsey A.R., Kitchin N., Absalon J., Gurtman A., Lockhart S., Neuzil K., Mulligan M.J., Bailey R. (2020). Safety and Immunogenicity of Two RNA-Based COVID-19 Vaccine Candidates. N. Engl. J. Med..

[24] Jackson L.A., Anderson E.J., Rouphael N.G., Roberts P.C., Makhene M., Coler R.N., McCullough M.P., Chappell J.D., Denison M.R., Stevens L.J. (2020). An mRNA Vaccine against SARS-CoV-2–Preliminary Report. N. Engl. J. Med..

[25] Wrapp D., Wang N., Corbett K.S., Goldsmith J.A., Hsieh C.L., Abiona O., Graham B.S., McLellan J.S. (2020). Cryo-EM structure of the 2019-nCoV spike in the prefusion conformation. Science.

[26] Mrak D., Simader E., Sieghart D., Mandl P., Radner H., Perkmann T., Haslacher H., Mayer M., Koblischke M., Hofer P. (2022). Immunogenicity and safety of a fourth COVID-19 vaccination in rituximab-treated patients: An open-label extension study. Ann. Rheum. Dis..

[27] Benjamini O., Gershon R., Bar-Haim E., Lustig Y., Cohen H., Doolman R., Kedmi M., Ribakovsky E., Kneller A., Hod T. (2023). Cellular and humoral response to the fourth BNT162b2 mRNA COVID-19 vaccine dose in patients with CLL. Eur. J. Haematol..

[28] Fendler A., Shepherd S.T.C., Au L., Wu M., Harvey R., Wilkinson K.A., Schmitt A.M., Tippu Z., Shum B., Farag S. (2022). Functional immune responses against SARS-CoV-2 variants of concern after fourth COVID-19 vaccine dose or infection in patients with blood cancer. Cell Rep. Med..

[29] Francis A.I., Ghany S., Gilkes T., Umakanthan S. (2022). Review of COVID-19 vaccine subtypes, efficacy and geographical distributions. Postgrad. Med. J..

[30] Rotshild V., Hirsh-Raccah B., Miskin I., Muszkat M., Matok I. (2021). Comparing the clinical efficacy of COVID-19 vaccines: A systematic review and network meta-analysis. Sci. Rep..

[31] Frenck R.W., Klein N.P., Kitchin N., Gurtman A., Absalon J., Lockhart S., Perez J.L., Walter E.B., Senders S., Bailey R. (2021). Safety, Immunogenicity, and Efficacy of the BNT162b2 COVID-19 Vaccine in Adolescents. N. Engl. J. Med..

[32] Ali K., Berman G., Zhou H., Deng W., Faughnan V., Coronado-Voges M., Ding B., Dooley J., Girard B., Hillebrand W. (2021). Evaluation of mRNA-1273 SARS-CoV-2 Vaccine in Adolescents. N. Engl. J. Med..

[33] Baden L.R., El Sahly H.M., Essink B., Kotloff K., Frey S., Novak R., Diemert D., Spector S.A., Rouphael N., Creech C.B. (2021). Efficacy and Safety of the mRNA-1273 SARS-CoV-2 Vaccine. N. Engl. J. Med..

[34] Sahin U., Muik A., Vogler I., Derhovanessian E., Kranz L.M., Vormehr M., Quandt J., Bidmon N., Ulges A., Baum A. (2021). BNT162b2 vaccine induces neutralizing antibodies and poly-specific T cells in humans. Nature.

[35] McDonald I., Murray S.M., Reynolds C.J., Altmann D.M., Boyton R.J. (2021). Comparative systematic review and meta-analysis of reactogenicity, immunogenicity and efficacy of vaccines against SARS-CoV-2. NPJ Vaccines.

[36] Kalimuddin S., Tham C.Y.L., Qui M., de Alwis R., Sim J.X.Y., Lim J.M.E., Tan H.C., Syenina A., Zhang S.L., Le Bert N. (2021). Early T cell and binding antibody responses are associated with COVID-19 RNA vaccine efficacy onset. Med.

[37] Rogliani P., Chetta A., Cazzola M., Calzetta L. (2021). SARS-CoV-2 Neutralizing Antibodies: A Network Meta-Analysis across Vaccines. Vaccines.

[38] Samanovic M.I., Cornelius A.R., Gray-Gaillard S.L., Allen J.R., Karmacharya T., Wilson J.P., Hyman S.W., Tuen M., Koralov S.B., Mulligan M.J. (2022). Robust immune responses are observed after one dose of BNT162b2 mRNA vaccine dose in SARS-CoV-2-experienced individuals. Sci. Transl. Med..

[39] Chagla Z. (2021). The BNT162b2 (BioNTech/Pfizer) vaccine had 95% efficacy against COVID-19 >/=7 days after the 2nd dose. Ann. Intern. Med..

[40] Lombardi A., Bozzi G., Ungaro R., Villa S., Castelli V., Mangioni D., Muscatello A., Gori A., Bandera A. (2021). Mini Review Immunological Consequences of Immunization With COVID-19 mRNA Vaccines: Preliminary Results. Front. Immunol..

[41] Gray A.N., Martin-Blais R., Tobin N.H., Wang Y., Brooker S.L., Li F., Gadoth A., Elliott J., Faure-Kumar E., Halbrook M. (2021). Humoral responses to SARS-CoV-2 mRNA vaccines: Role of past infection. PLoS ONE.

[42] Echaide M., Labiano I., Delgado M., Fernandez de Lascoiti A., Ochoa P., Garnica M., Ramos P., Chocarro L., Fernandez L., Arasanz H. (2022). Immune Profiling Uncovers Memory T-Cell Responses with a Th17 Signature in Cancer Patients with Previous SARS-CoV-2 Infection Followed by mRNA Vaccination. Cancers.

[43] Sheng W.H., Ieong S.M., Lin P.H., Hsieh M.J., Yang H.C., Pan C.F., Chao T.L., Chang S.Y., Chang S.C. (2023). Immunogenicity and safety of third-dose mRNA COVID-19 vaccines in healthy adults previously vaccinated with two doses of the ChAdOx1 vaccine. J. Formos. Med. Assoc..

[44] Canetti M., Barda N., Gilboa M., Indenbaum V., Asraf K., Gonen T., Weiss-Ottolenghi Y., Amit S., Doolman R., Mendelson E. (2022). Six-Month Follow-up after a Fourth BNT162b2 Vaccine Dose. N. Engl. J. Med..

[45] Moreira E.D., Kitchin N., Xu X., Dychter S.S., Lockhart S., Gurtman A., Perez J.L., Zerbini C., Dever M.E., Jennings T.W. (2022). Safety and Efficacy of a Third Dose of BNT162b2 COVID-19 Vaccine. N. Engl. J. Med..

[46] Korth J., Jahn M., Dorsch O., Anastasiou O.E., Sorge-Hadicke B., Eisenberger U., Gackler A., Dittmer U., Witzke O., Wilde B. (2021). Impaired Humoral Response in Renal Transplant Recipients to SARS-CoV-2 Vaccination with BNT162b2 (Pfizer-BioNTech). Viruses.

[47] Lerner A.H., Arvanitis P., Vieira K., Klein E.J., Farmakiotis D. (2022). mRNA Vaccination Decreases COVID-19-Associated Morbidity and Mortality Among Organ Transplant Recipients: A Contemporary Cohort Study. Open Forum Infect. Dis..

[48] Thomson T., Prendecki M., Gleeson S., Martin P., Spensley K., De Aguiar R.C., Sandhu B., Seneschall C., Gan J., Clarke C.L. (2022). Immune responses following 3rd and 4th doses of heterologous and homologous COVID-19 vaccines in kidney transplant recipients. eClinicalMedicine.

[49] Infantino M., Tsalouchos A., Russo E., Laudicina S., Grossi V., Lari B., Benucci M., Stacchini L., Amedei A., Casprini P. (2022). Assessing T-Cell Immunity in Kidney Transplant Recipients with Absent Antibody Production after a 3rd Dose of the mRNA-1273 Vaccine. Int. J. Mol. Sci..

[50] Barczi E., Varga V., Nagy A., Eszes N., Jaky-Kovats Z., Muller V., Bohacs A. (2022). Serological findings following the second and third SARS-CoV-2 vaccines in lung transplant recipients. Immun. Inflamm. Dis..

[51] Maniscalco G.T., Scavone C., Mascolo A., Manzo V., Prestipino E., Guglielmi G., Aiezza M.L., Cozzolino S., Bracco A., Moreggia O. (2022). The Safety Profile of COVID-19 Vaccines in Patients Diagnosed with Multiple Sclerosis: A Retrospective Observational Study. J. Clin. Med..

[52] Maniscalco G.T., Ferrara A.L., Liotti A., Manzo V., Di Battista M.E., Salvatore S., Graziano D., Viola A., Amato G., Moreggia O. (2022). Long term persistence of SARS-CoV-2 humoral response in multiple sclerosis subjects. Mult. Scler. Relat. Disord..

[53] Alfonso-Dunn R., Lin J., Kirschner V., Lei J., Feuer G., Malin M., Liu J., Roche M., Sadiq S.A. (2022). Strong T-cell activation in response to COVID-19 vaccination in multiple sclerosis patients receiving B-cell depleting therapies. Front. Immunol..

[54] Zaleska J., Kwasnik P., Paziewska M., Purkot J., Szabelak A., Jurek M., Masny N., Dziatkiewicz I., Pronobis-Szczylik B., Piebiak A. (2023). Response to anti-SARS-CoV-2 mRNA vaccines in multiple myeloma and chronic lymphocytic leukemia patients. Int. J. Cancer.

[55] De Placido P., Pietroluongo E., De Angelis C., Tafuro M., Barraco C., Giannatiempo R., Buonaiuto R., Schettini F., Iervolino A., Vozzella E.A. (2022). Safety and immunogenicity of the COVID-19 vaccine BNT162b2 for patients with breast and gynecological cancer on active anticancer therapy: Results of a prospective observational study. Front. Oncol..

[56] Ligumsky H., Safadi E., Etan T., Vaknin N., Waller M., Croll A., Nikolaevski-Berlin A., Greenberg I., Halperin T., Wasserman A. (2022). Immunogenicity and Safety of the BNT162b2 mRNA COVID-19 Vaccine Among Actively Treated Cancer Patients. J. Natl. Cancer Inst..

[57] Bagacean C., Letestu R., Al-Nawakil C., Brichler S., Levy V., Sritharan N., Delmer A., Dartigeas C., Leblond V., Roos-Weil D. (2022). Humoral response to mRNA anti-COVID-19 vaccines BNT162b2 and mRNA-1273 in patients with chronic lymphocytic leukemia. Blood Adv..

[58] Kang W., Shami J.J.P., Yan V.K.C., Ye X., Blais J.E., Li X., Lee V.H.F., Chui C.S.L., Lai F.T.T., Wan E.Y.F. (2022). Safety of two-dose COVID-19 vaccination (BNT162b2 and CoronaVac) in adults with cancer: A territory-wide cohort study. J. Hematol. Oncol..

[59] Yamasaki E., Shimamoto F., Nishikawa H., Goto M., Iwamoto M., Kimura K., Ukimura A., Oosaka N., Taniguchi K., Ono F. (2022). A Prospective Study Regarding the Efficacy and Safety of the BNT162b2 Vaccine in Patients With Solid Malignancies Undergoing Systemic Chemotherapy. In Vivo.

[60] Diamantopoulos P.T., Kontandreopoulou C.N., Stafylidis C., Vlachopoulou D., Giannakopoulou N., Vardaka M., Mpouhla A., Variami E., Galanopoulos A., Pappa V. (2022). Immunogenicity of a third dose of the BNT162b2 COVID-19 vaccine in patients with CLL: Effects on treatment selection. Ann. Hematol..

[61] Goel R.R., Painter M.M., Apostolidis S.A., Mathew D., Meng W., Rosenfeld A.M., Lundgreen K.A., Reynaldi A., Khoury D.S., Pattekar A. (2021). mRNA vaccines induce durable immune memory to SARS-CoV-2 and variants of concern. Science.

[62] Harrington P., Doores K.J., Radia D., O’Reilly A., Lam H.P.J., Seow J., Graham C., Lechmere T., McLornan D., Dillon R. (2021). Single dose of BNT162b2 mRNA vaccine against severe acute respiratory syndrome coronavirus-2 (SARS-CoV-2) induces neutralising antibody and polyfunctional T-cell responses in patients with chronic myeloid leukaemia. Br. J. Haematol..

[63] Monin L., Laing A.G., Munoz-Ruiz M., McKenzie D.R., Del Molino Del Barrio I., Alaguthurai T., Domingo-Vila C., Hayday T.S., Graham C., Seow J. (2021). Safety and immunogenicity of one versus two doses of the COVID-19 vaccine BNT162b2 for patients with cancer: Interim analysis of a prospective observational study. Lancet Oncol..

[64] Ehmsen S., Asmussen A., Jeppesen S.S., Nilsson A.C., Osterlev S., Vestergaard H., Justesen U.S., Johansen I.S., Frederiksen H., Ditzel H.J. (2021). Antibody and T cell immune responses following mRNA COVID-19 vaccination in patients with cancer. Cancer Cell.

[65] Mastroianni F., Guida P., Bellanova G., Valentina De Nicolo E., Righetti G., Formoso M., Celani F. (2022). SARS-CoV-2 antibody response after BNT162b2 mRNA vaccine in healthcare workers: Nine-month of follow-up. Vaccine X.

[66] Wand O., Breslavsky A., Bar-Shai A., Levy C., Maayan S., Rimler A., Zwahra M., Cohen-Hagai K., Harish A., Zacks N. (2023). One-year dynamics of antibody titers after three doses of SARS-CoV-2 BNT162b2 vaccine. Vaccine.

[67] Herishanu Y., Avivi I., Levi S., Shefer G., Bronstein Y., Moshiashvili M.M., Ziv T., Scarfo L., Perry C., Ghia P. (2022). Six-month antibody persistence after BNT162b2 mRNA COVID-19 vaccination in patients with chronic lymphocytic leukemia. Blood Adv..

[68] Vollenberg R., Tepasse P.R., Kuhn J.E., Hennies M., Strauss M., Rennebaum F., Schomacher T., Boeckel G., Lorentzen E., Bokemeyer A. (2022). Humoral Immune Response in IBD Patients Three and Six Months after Vaccination with the SARS-CoV-2 mRNA Vaccines mRNA-1273 and BNT162b2. Biomedicines.

[69] Guerrera G., Picozza M., D’Orso S., Placido R., Pirronello M., Verdiani A., Termine A., Fabrizio C., Giannessi F., Sambucci M. (2021). BNT162b2 vaccination induces durable SARS-CoV-2-specific T cells with a stem cell memory phenotype. Sci. Immunol..

[70] Mahnke Y.D., Brodie T.M., Sallusto F., Roederer M., Lugli E. (2013). The who’s who of T-cell differentiation: Human memory T-cell subsets. Eur. J. Immunol..

[71] Lanna A., Gomes D.C., Muller-Durovic B., McDonnell T., Escors D., Gilroy D.W., Lee J.H., Karin M., Akbar A.N. (2017). A sestrin-dependent Erk-Jnk-p38 MAPK activation complex inhibits immunity during aging. Nat. Immunol..

[72] Lanna A., Henson S.M., Escors D., Akbar A.N. (2014). The kinase p38 activated by the metabolic regulator AMPK and scaffold TAB1 drives the senescence of human T cells. Nat. Immunol..

[73] Egwuagu C.E. (2009). STAT3 in CD4+ T helper cell differentiation and inflammatory diseases. Cytokine.

[74] Trapnell B.C., Carey B.C., Uchida K., Suzuki T. (2009). Pulmonary alveolar proteinosis, a primary immunodeficiency of impaired GM-CSF stimulation of macrophages. Curr. Opin. Immunol..

[75] Supriya R., Gao Y., Gu Y., Baker J.S. (2021). Role of Exercise Intensity on Th1/Th2 Immune Modulations During the COVID-19 Pandemic. Front. Immunol..

[76] Martonik D., Parfieniuk-Kowerda A., Rogalska M., Flisiak R. (2021). The Role of Th17 Response in COVID-19. Cells.

[77] Lonberg N. (2008). Fully human antibodies from transgenic mouse and phage display platforms. Curr. Opin. Immunol..

[78] Gandolfo C., Anichini G., Mugnaini M., Bocchia M., Terrosi C., Sicuranza A., Gori Savellini G., Gozzetti A., Franchi F., Cusi M.G. (2022). Overview of Anti-SARS-CoV-2 Immune Response Six Months after BNT162b2 mRNA Vaccine. Vaccines.

[79] Arasanz H., Bocanegra A.I., Morilla I., Fernandez-Irigoyen J., Martinez-Aguillo M., Teijeira L., Garnica M., Blanco E., Chocarro L., Ausin K. (2022). Circulating Low Density Neutrophils Are Associated with Resistance to First Line Anti-PD1/PDL1 Immunotherapy in Non-Small Cell Lung Cancer. Cancers.

[80] Bocanegra A., Fernandez G., Ajona D., Arasanz H., Blanco E., Zuazo M., Chocarro L., Pineiro-Hermida S., Morente P., Fernandez L. (2022). Potent clinical predictive and systemic adjuvant therapeutic value of plasma fractalkine in PD-L1/PD-1 blockade immunotherapy for lung cancer. MedRxiv.

[81] Schultze J.L., Mass E., Schlitzer A. (2019). Emerging Principles in Myelopoiesis at Homeostasis and during Infection and Inflammation. Immunity.

[82] He Y.M., Li X., Perego M., Nefedova Y., Kossenkov A.V., Jensen E.A., Kagan V., Liu Y.F., Fu S.Y., Ye Q.J. (2018). Transitory presence of myeloid-derived suppressor cells in neonates is critical for control of inflammation. Nat. Med..

[83] Diakos C.I., Charles K.A., McMillan D.C., Clarke S.J. (2014). Cancer-related inflammation and treatment effectiveness. Lancet. Oncol..

[84] Sevko A., Sade-Feldman M., Kanterman J., Michels T., Falk C.S., Umansky L., Ramacher M., Kato M., Schadendorf D., Baniyash M. (2013). Cyclophosphamide promotes chronic inflammation-dependent immunosuppression and prevents antitumor response in melanoma. J. Investig. Derm..

[85] Sevko A., Michels T., Vrohlings M., Umansky L., Beckhove P., Kato M., Shurin G.V., Shurin M.R., Umansky V. (2013). Antitumor effect of paclitaxel is mediated by inhibition of myeloid-derived suppressor cells and chronic inflammation in the spontaneous melanoma model. J. Immunol..

[86] Sanchez C.M., Gebauer F., Sune C., Mendez A., Dopazo J., Enjuanes L. (1992). Genetic evolution and tropism of transmissible gastroenteritis coronaviruses. Virology.

[87] Lan J., Ge J., Yu J., Shan S., Zhou H., Fan S., Zhang Q., Shi X., Wang Q., Zhang L. (2020). Structure of the SARS-CoV-2 spike receptor-binding domain bound to the ACE2 receptor. Nature.

[88] Weissman D., Alameh M.G., de Silva T., Collini P., Hornsby H., Brown R., LaBranche C.C., Edwards R.J., Sutherland L., Santra S. (2021). D614G Spike Mutation Increases SARS CoV-2 Susceptibility to Neutralization. Cell Host Microbe.

[89] Zou J., Xie X., Fontes-Garfias C.R., Swanson K.A., Kanevsky I., Tompkins K., Cutler M., Cooper D., Dormitzer P.R., Shi P.Y. (2021). The effect of SARS-CoV-2 D614G mutation on BNT162b2 vaccine-elicited neutralization. NPJ Vaccines.

[90] Wang Z., Schmidt F., Weisblum Y., Muecksch F., Barnes C.O., Finkin S., Schaefer-Babajew D., Cipolla M., Gaebler C., Lieberman J.A. (2021). mRNA vaccine-elicited antibodies to SARS-CoV-2 and circulating variants. Nature.

[91] Wang P., Nair M.S., Liu L., Iketani S., Luo Y., Guo Y., Wang M., Yu J., Zhang B., Kwong P.D. (2021). Antibody resistance of SARS-CoV-2 variants B.1.351 and B.1.1.7. Nature.

[92] Madhi S.A., Baillie V., Cutland C.L., Voysey M., Koen A.L., Fairlie L., Padayachee S.D., Dheda K., Barnabas S.L., Bhorat Q.E. (2021). Efficacy of the ChAdOx1 nCoV-19 COVID-19 Vaccine against the B.1.351 Variant. N. Engl. J. Med..

[93] Ren S.Y., Wang W.B., Gao R.D., Zhou A.M. (2022). Omicron variant (B.1.1.529) of SARS-CoV-2: Mutation, infectivity, transmission, and vaccine resistance. World J. Clin. Cases.

[94] Chavda V.P., Apostolopoulos V. (2022). Global impact of delta plus variant and vaccination. Expert Rev. Vaccines.

[95] Khan A., Zia T., Suleman M., Khan T., Ali S.S., Abbasi A.A., Mohammad A., Wei D.Q. (2021). Higher infectivity of the SARS-CoV-2 new variants is associated with K417N/T, E484K, and N501Y mutants: An insight from structural data. J. Cell. Physiol..

[96] Lopez Bernal J., Andrews N., Gower C., Gallagher E., Simmons R., Thelwall S., Stowe J., Tessier E., Groves N., Dabrera G. (2021). Effectiveness of COVID-19 Vaccines against the B.1.617.2 (Delta) Variant. N. Engl. J. Med..

[97] Fiolet T., Kherabi Y., MacDonald C.J., Ghosn J., Peiffer-Smadja N. (2022). Comparing COVID-19 vaccines for their characteristics, efficacy and effectiveness against SARS-CoV-2 and variants of concern: A narrative review. Clin. Microbiol. Infect..

[98] Dhawan M., Sharma A., Priyanka, Thakur N., Rajkhowa T.K., Choudhary O.P. (2022). Delta variant (B.1.617.2) of SARS-CoV-2: Mutations, impact, challenges and possible solutions. Hum. Vaccines Immunother..

[99] Christie S.M., Tada T., Yin Y., Bhardwaj A., Landau N.R., Rothenberg E. (2022). Single-virus tracking reveals variant SARS-CoV-2 spike proteins induce ACE2-independent membrane interactions. Sci. Adv..

[100] Sanchez C.M., Pascual-Iglesias A., Sola I., Zuniga S., Enjuanes L. (2019). Minimum Determinants of Transmissible Gastroenteritis Virus Enteric Tropism Are Located in the N-Terminus of Spike Protein. Pathogens.

[101] Enjuanes L., Sanchez C., Gebauer F., Mendez A., Dopazo J., Ballesteros M.L. (1993). Evolution and tropism of transmissible gastroenteritis coronavirus. Adv. Exp. Med. Biol..

[102] Ballesteros M.L., Sanchez C.M., Martin-Caballero J., Enjuanes L. (1995). Molecular bases of tropism in the PUR46 cluster of transmissible gastroenteritis coronaviruses. Adv. Exp. Med. Biol..

[103] Grune B., Grune J., Kossow A., Joisten C. (2022). Vaccination and Transmission Risk during the Outbreak of B.1.1.529 (Omicron). Vaccines.

[104] Shah M., Woo H.G. (2021). Omicron: A Heavily Mutated SARS-CoV-2 Variant Exhibits Stronger Binding to ACE2 and Potently Escapes Approved COVID-19 Therapeutic Antibodies. Front. Immunol..

[105] Zhao M.M., Zhu Y., Zhang L., Zhong G., Tai L., Liu S., Yin G., Lu J., He Q., Li M.J. (2022). Novel cleavage sites identified in SARS-CoV-2 spike protein reveal mechanism for cathepsin L-facilitated viral infection and treatment strategies. Cell Discov..

[106] Tulimilli S.V., Dallavalasa S., Basavaraju C.G., Kumar Rao V., Chikkahonnaiah P., Madhunapantula S.V., Veeranna R.P. (2022). Variants of Severe Acute Respiratory Syndrome Coronavirus 2 (SARS-CoV-2) and Vaccine Effectiveness. Vaccines.

[107] Goh Y.S., Rouers A., Fong S.W., Zhuo N.Z., Hor P.X., Loh C.Y., Huang Y., Neo V.K., Kam I.K.J., Wang B. (2022). Waning of specific antibodies against Delta and Omicron variants five months after a third dose of BNT162b2 SARS-CoV-2 vaccine in elderly individuals. Front. Immunol..

[108] Qu P., Evans J.P., Faraone J.N., Zheng Y.M., Carlin C., Anghelina M., Stevens P., Fernandez S., Jones D., Lozanski G. (2023). Enhanced neutralization resistance of SARS-CoV-2 Omicron subvariants BQ.1, BQ.1.1, BA.4.6, BF.7, and BA.2.75.2. Cell Host Microbe.

[109] Gupta S.L., Mantus G., Manning K.E., Ellis M., Patel M., Ciric C.R., Lu A., Turner J.S., O’Halloran J.A., Presti R.M. (2022). Loss of Pfizer (BNT162b2) Vaccine-Induced Antibody Responses against the SARS-CoV-2 Omicron Variant in Adolescents and Adults. J. Virol..

[110] Zou Y., Huang D., Jiang Q., Guo Y., Chen C. (2022). The Vaccine Efficacy Against the SARS-CoV-2 Omicron: A Systemic Review and Meta-Analysis. Front. Public Health.

[111] Zhang G.F., Meng W., Chen L., Ding L., Feng J., Perez J., Ali A., Sun S., Liu Z., Huang Y. (2022). Neutralizing antibodies to SARS-CoV-2 variants of concern including Delta and Omicron in subjects receiving mRNA-1273, BNT162b2, and Ad26.COV2.S vaccines. J. Med. Virol..

[112] Chatzilena A., Hyams C., Challen R., Marlow R., King J., Adegbite D., Kinney J., Clout M., Maskell N., Oliver J. (2023). Effectiveness of BNT162b2 COVID-19 vaccination in prevention of hospitalisations and severe disease in adults with SARS-CoV-2 Delta (B.1.617.2) and Omicron (B.1.1.529) variant between June 2021 and July 2022: A prospective test negative case-control study. Lancet Reg. Health Eur..

[113] Wang Q., Iketani S., Li Z., Liu L., Guo Y., Huang Y., Bowen A.D., Liu M., Wang M., Yu J. (2023). Alarming antibody evasion properties of rising SARS-CoV-2 BQ and XBB subvariants. Cell.

[114] (2022). COVID-19 update: Bivalent Pfizer and Moderna vaccines authorized for children >/=6 months old. Med. Lett. Drugs Ther..

[115] Chalkias S., Harper C., Vrbicky K., Walsh S.R., Essink B., Brosz A., McGhee N., Tomassini J.E., Chen X., Chang Y. (2022). A Bivalent Omicron-Containing Booster Vaccine against COVID-19. N. Engl. J. Med..

[116] Winokur P., Gayed J., Fitz-Patrick D., Thomas S.J., Diya O., Lockhart S., Xu X., Zhang Y., Bangad V., Schwartz H.I. (2023). Bivalent Omicron BA.1-Adapted BNT162b2 Booster in Adults Older than 55 Years. N. Engl. J. Med..

[117] Patone M., Mei X.W., Handunnetthi L., Dixon S., Zaccardi F., Shankar-Hari M., Watkinson P., Khunti K., Harnden A., Coupland C.A.C. (2022). Risks of myocarditis, pericarditis, and cardiac arrhythmias associated with COVID-19 vaccination or SARS-CoV-2 infection. Nat. Med..

[118] Patone M., Mei X.W., Handunnetthi L., Dixon S., Zaccardi F., Shankar-Hari M., Watkinson P., Khunti K., Harnden A., Coupland C.A.C. (2022). Risk of Myocarditis After Sequential Doses of COVID-19 Vaccine and SARS-CoV-2 Infection by Age and Sex. Circulation.

[119] Naveed Z., Li J., Wilton J., Spencer M., Naus M., Velasquez Garcia H.A., Kwong J.C., Rose C., Otterstatter M., Janjua N.Z. (2022). Comparative Risk of Myocarditis/Pericarditis Following Second Doses of BNT162b2 and mRNA-1273 Coronavirus Vaccines. J. Am. Coll. Cardiol..

[120] Wong H.L., Hu M., Zhou C.K., Lloyd P.C., Amend K.L., Beachler D.C., Secora A., McMahill-Walraven C.N., Lu Y., Wu Y. (2022). Risk of myocarditis and pericarditis after the COVID-19 mRNA vaccination in the USA: A cohort study in claims databases. Lancet.

[121] Lessans N., Rottenstreich A., Stern S., Gilan A., Saar T.D., Porat S., Dior U.P. (2023). The effect of BNT162b2 SARS-CoV-2 mRNA vaccine on menstrual cycle symptoms in healthy women. Int. J. Gynaecol. Obstet..

[122] Dellino M., Lamanna B., Vinciguerra M., Tafuri S., Stefanizzi P., Malvasi A., Di Vagno G., Cormio G., Loizzi V., Cazzato G. (2022). SARS-CoV-2 Vaccines and Adverse Effects in Gynecology and Obstetrics: The First Italian Retrospective Study. Int. J. Environ. Res. Public Health.

[123] Callaway E. (2023). The next generation of coronavirus vaccines: A graphical guide. Nature.

[124] Azim Majumder M.A., Razzaque M.S. (2022). Repeated vaccination and ‘vaccine exhaustion’: Relevance to the COVID-19 crisis. Expert Rev. Vaccines.

[125] Benitez Fuentes J.D., Mohamed Mohamed K., de Luna Aguilar A., Jimenez Garcia C., Guevara-Hoyer K., Fernandez-Arquero M., Rodriguez de la Pena M.A., Garciia Bravo L., Jimenez Ortega A.F., Flores Navarro P. (2022). Evidence of exhausted lymphocytes after the third anti-SARS-CoV-2 vaccine dose in cancer patients. Front. Oncol..

[126] Abavisani M., Rahimian K., Mahdavi B., Tokhanbigli S., Mollapour Siasakht M., Farhadi A., Kodori M., Mahmanzar M., Meshkat Z. (2022). Mutations in SARS-CoV-2 structural proteins: A global analysis. Virol. J..

